# Elderly patients with stage II gastric cancer do not benefit from adjuvant chemotherapy

**DOI:** 10.1186/s12957-023-03185-5

**Published:** 2023-10-11

**Authors:** Jianping Guo, Zhizhong Xiong, Shi Yin, Yue’e Wen, Longyang Jin, Caiqin Wang, Huaxian Chen, Dandong Luo, Zijian Deng, Dayin Huang, Xianzhe Li, Biying Yi, Chaobin Mao, Lei Lian

**Affiliations:** 1 Department of General Surgery (Department of Gastrointestinal Surgery), The Sixth Affiliated Hospital, Sun Yat-Sen University, Guangzhou, China; 2 Guangdong Provincial Key Laboratory of Colorectal and Pelvic Floor Diseases, The Sixth Affiliated Hospital, Sun Yat-Sen University, Guangzhou, China; 3 Biomedical Innovation Center, The Sixth Affiliated Hospital, Sun Yat-Sen University, Guangzhou, China; 4 Follow-up office of the Sixth Affiliated Hospital, The Sixth Affiliated Hospital, Sun Yat-sen University, Guanzhou, China

**Keywords:** Gastric cancer, Elderly, Adjuvant chemotherapy, Survival

## Abstract

**Background:**

With the aging of the population, the burden of elderly gastric cancer (EGC) increases worldwide. However, there is no consensus on the definition of EGC and the efficacy of adjuvant chemotherapy in patients with stage II EGC. Here, we investigated the effectiveness of adjuvant chemotherapy in defined EGC patients.

**Methods:**

We enrolled 5762 gastric cancer patients of three independent cohorts from the Sixth Affiliated Hospital of Sun Yat-sen University (local), the Surveillance, Epidemiology, and End Results (SEER), and the Asian Cancer Research Group (ACRG). The optimal age cutoff for EGC was determined using the K-adaptive partitioning algorithm. The defined EGC group and the efficacy of adjuvant chemotherapy for them were confirmed by Cox regression and Kaplan–Meier survival analyses. Furthermore, gene set variation analyses (GSVA) were performed to reveal pathway enrichment between groups.

**Results:**

The optimal age partition value for EGC patients was 75. In the local, SEER, and ACRG cohorts, the EGC group exhibited significantly worse overall survival and cancer-specific survival than the non-EGC group (*P* < 0.05) and was an independent risk factor. Stratified analyses based on chemotherapy showed that EGC patients derived little benefit from adjuvant chemotherapy. Furthermore, GSVA analysis revealed the activation of DNA repair-related pathways and downregulation of the p53 pathway, which may partially contribute to the observed findings.

**Conclusion:**

In this retrospective, international multi-center study, 75 years old was identified as the optimal age cutoff for EGC definition, and adjuvant chemotherapy proved to be unbeneficial for stage II EGC patients.

**Supplementary Information:**

The online version contains supplementary material available at 10.1186/s12957-023-03185-5.

## Background

As the fourth largest cause of cancer mortality globally, gastric cancer (GC) constitutes a major threat to global public health, especially to the elderly, who account for more than 70% of GC-related mortality [1]. In light of the aging population, the proportion of elderly gastric cancer (EGC) patients will increase tremendously due to the longer life expectancy [2]. Even though it is generally accepted that the elderly group has a particularly poor prognosis, a unified threshold defining EGC patients has not yet been reached and needs to be resolved [3].

Another issue to clarify is adjuvant chemotherapy effectiveness in elderly patients with stage II GC. As the National Comprehensive Cancer Network (NCCN) recommended, adjuvant chemotherapy should be performed in all stage II GC patients, especially those with a high risk of recurrence [4]. However, current guidelines are primarily based on clinical trials in patients younger than 75 years old because old individuals are commonly excluded from these trials [2, 5]. Besides, compared to the younger patients, EGC patients are generally in worse health, such as higher comorbidities incidence, higher postoperative complication chance, and shorter life expectancies. They may therefore be at a higher risk of chemotherapy-related morbidity and mortality [6–8]. Whether adjuvant chemotherapy benefits stage II EGC patients after radical gastrectomy is still debated.

Here, based on three international multi-center GC cohorts, we explored the optimal age cutoff for EGC definition in the derivation cohorts and further investigated the prognostic value of adjuvant chemotherapy for newly defined EGC patients of the internal and external cohorts, with an aim to bring a novel insight into the treatment and clinical feature of EGC.

## Materials and methods

### Patient selection

The study included three independent GC patient cohorts: the local cohort (*n* = 626), the Surveillance, Epidemiology, and End Results (SEER) cohort (*n* = 5040), and the Asian Cancer Research Group (ACRG) cohort (*n* = 96). The local and SEER cohorts, the initial derivation cohorts, consisted of patients diagnosed with primary GC patients from the Sixth Affiliated Hospital of Sun Yat-sen University (SAH-SYSU) from August 2008 to August 2021 and the SEER database from January 2010 to December 2018, which is maintained by the National Cancer Institute and comprises comprehensive cancer data collected from 18 different regions or states across the USA. We accessed and extracted the relevant data from the SEER database using SEER*Stat version 8.3.6 (http://seer.cancer.gov/seerstat/). All data for this study was collected retrospectively, and the inclusion criteria were diagnosed with primary gastric adenocarcinoma, stages II–III adhering to the American Joint Committee on Cancer (AJCC) 8th stage system, and received gastrectomy. Moreover, patients with short follow-up (less than three months), unknown survival status, and other inadequate clinicopathological data shown in Table 1 were excluded from this study. As shown in Fig. S1, the ACRG cohort (Accession number: GSE62254) was obtained from the Gene Expression Omnibus (GEO) on 15 June 2022 as an external validation cohort.

**Table 1:** The clinicopathological characteristics in stage II elderly GC patients of local cohort and SEER cohort

Characteristic	Local cohort			SEER cohort		
	Non-EGC (*N* = 283)	EGC (*N* = 21)	*p* value^a^	Non-EGC (*N* = 909)	EGC (*N* = 423)	*p* value^a^
Gender			0.211			0.001
Male	184 (65.0%)	17 (81.0%)		560 (61.6%)	220 (52.0%)	
Female	99 (35.0%)	4 (19.0%)		349 (38.4%)	203 (48.0%)	
BMI (kg/m^2^)			< 0.001			
18.5–24	190 (67.1%)	10 (47.6%)				
< 18.5	26 (9.2%)	9 (42.9%)				
≥ 24	67 (23.7%)	2 (9.5%)				
CEA			0.615			
Negative	238 (84.1%)	15 (71.4%)				
Positive	45 (15.9%)	6 (28.6%)				
CA199			0.015			
Negative	257 (90.8%)	15 (71.4%)				
Positive	26 (9.2%)	6 (28.6%)				
Location			0.499			0.015
Lower	110 (38.9%)	7 (33.3%)		372 (40.9%)	210 (49.6%)	
Upper	93 (32.9%)	10 (47.6%)		47 (5.2%)	13 (3.1%)	
Middle	71 (25.1%)	4 (19.0%)		405 (44.6%)	168 (39.7%)	
Overlapped	9 (3.2%)	0 (0%)		85 (9.4%)	32 (7.6%)	
Histology						< 0.001
Adenocarcinoma				666 (73.3%)	383 (90.5%)	
Signet ring cell carcinoma				243 (26.7%)	40 (9.5%)	
Lauren type			0.111			
Intestinal	89 (31.4%)	8 (38.1%)				
Diffuse	117 (41.3%)	4 (19.0%)				
Mixed	77 (27.2%)	9 (42.9%)				
Grade			0.502			0.017
Well	16 (5.7%)	0 (0%)		25 (2.8%)	16 (3.8%)	
Moderately	86 (30.4%)	6 (28.6%)		274 (30.1%)	157 (37.1%)	
Poorly and undifferentiated	181 (64.0%)	15 (71.4%)		610 (67.1%)	250 (59.1%)	
T stage			0.989			0.353
T1-2	48 (17.0%)	3 (14.3%)		227 (25.0%)	95 (22.5%)	
T3-4	235 (83.0%)	18 (85.7%)		682 (75.0%)	328 (77.5%)	
N stage			0.950			0.182
N0	149 (52.7%)	12 (57.1%)		419 (46.1%)	220 (52.0%)	
N1	110 (38.9%)	7 (33.3%)		370 (40.7%)	155 (36.6%)	
N2	23 (8.1%)	2 (9.5%)		104 (11.4%)	44 (10.4%)	
N3	1 (0.4%)	0 (0%)		16 (1.8%)	4 (0.9%)	
Surgery			0.163			
Open	86 (30.4%)	10 (47.6%)				
Laparoscope	197 (69.6%)	11 (52.4%)				
Chemotherapy			0.064			< 0.001
No	53 (18.7%)	8 (38.1%)		212 (23.3%)	268 (63.4%)	
Yes	230 (81.3%)	13 (61.9%)		697 (76.7%)	155 (36.6%)	

^a^Chi-square test or Fisher’s exact test

All patients received radical gastrectomy for the local cohort and were staged through histopathological examination based on the AJCC 8th stage system. Preoperative data was measured within 2 weeks before surgery, including body weight, carcinoembryonic antigen (CEA), and carbohydrate antigen (CA) 199. Adjuvant chemotherapy was performed after surgery, and its regimens include both monotherapy and combination therapy. For monotherapy, it consists of an oral S-1 regimen. As combination therapy, it includes oral S-1 with intravenous oxaliplatin (SOX), oral capecitabine with intravenous oxaliplatin (XELOX), or intravenous oxaliplatin, leucovorin, and 5-fluorouracil (FOLFOX) regimens. Each regimen is dosed according to the NCCN guidelines and administered for at least four cycles. For SEER and ACRG cohorts, analytic variables were derived directly from the relative fields of the public dataset based on the corresponding documentation for reference. This study was approved by the Clinical Research Ethics Committee of the Sixth Affiliated Hospital of Sun Yat-sen University.

### Statistical analysis

First, the K-adaptive partitioning for survival data (KAPS) algorithm, an unsupervised approach for prognosis partitioning [9], was performed to choose the optimal cutoff for EGC definition based on the initial local and SEER patients with stage II–III GC. Then, divided by the age cutoff, the stage II local and SEER cohorts were included in subsequent analyses. All qualitative data were presented as proportions and analyzed using the chi-square test or Fisher’s exact test. Overall survival (OS) and cancer-specific survival (CSS) were the main outcome variables of this study and were described as survival months after surgery. Log-rank test and Kaplan–Meier method were used to assess these survivals. Besides, to identify independent prognostic factors, multivariate Cox regression was applied to all significant variables (*P* less than 0.1) on univariate Cox regression. Moreover, a chemotherapy-based stratified log-rank test was conducted to evaluate the significance of adjuvant chemotherapy in stage II EGC patients. Similar analyses were reperformed in the external validation cohort. Finally, based on the molecular signatures database (Hallmark and KEGG collections), gene set variation analyses (GSVA) were implemented to calculate sample-wise enrichment scores for related gene sets by the “GSVA” package, which is a computational method that evaluates the activity or enrichment of gene sets or pathways in gene expression data, enabling the inference of functional changes in biological processes or pathways across different age conditions. The GSVA scores were visualized by the “pheatmap” package. The correlation between diagnostic age and pathway GSVA scores was assessed by Pearson correlation coefficients to determine age-related gene sets (*P* < 0.05 and |coefficient|> 0.2), which was associated with bad prognosis in EGC patients.

All statistical analyses were performed in R software (R project, Version 4.1.2). For all tests, *P* < 0.05 (two-tailed) was statistically significant.

## Results

### Determine optimal age cutoff

**Fig. 1:**
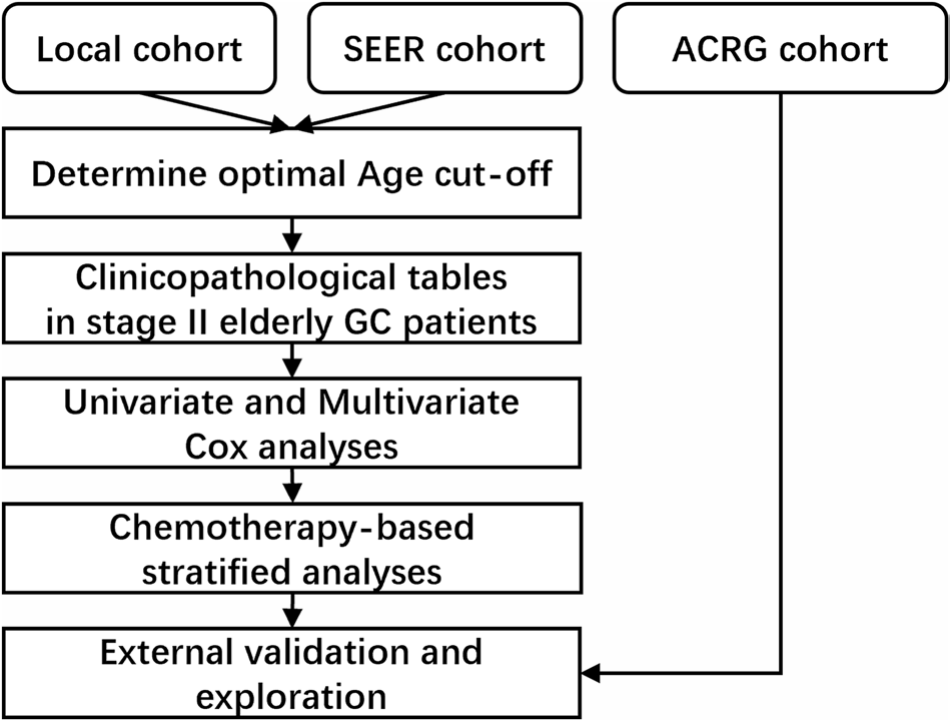
Flow diagram of this study

As the study flow diagram is represented in Fig. 1, first, we included 5666 patients with stage II-III primary GC (626 in the local cohort and 5040 in the SEER cohort) as the derivation cohort. Then, the optimal age cutoff was determined to define the EGC patients. Using the KAPS algorithm to divide each derivation cohort into two subgroups based on death incidence, we found the optimal age partition values of EGC patients were 75 and 77 in the local cohort and SEER cohort, which were very close (Fig. S2 A, D). To make the partition values easier to remember, we set 75 as the optimal age cutoff. As depicted in Fig. S2, OS and CSS in stage II–III GC patients aged ≥ 75 years were both significantly worse than patients aged < 75 years, whether in the local or SEER cohort (*P* < 0.05 for all log-rank tests).

### Clinicopathological tables in stage II elderly GC patients

The mean age of stage II GC patients in local and SEER cohorts who fulfilled the inclusion criteria was 61 (range, 21 to 85) and 68 (range, 19 to 98). The mean follow-up duration of local and SEER cohorts was 33.55 months (range, 3.32 to 109.85 months) and 46.00 months (range, 3.00 to 107.00 months).

Accordingly, we identified two subgroups of stage II GC patients as EGC patients (aged ≥ 75) and non-EGC patients (aged < 75). 21 of 304 patients in the local cohort and 423 of 1332 in the SEER cohort were defined as EGC. The clinicopathologic features in the local and SEER cohorts are listed in Table 1. The EGC group exhibited lower body mass index (BMI) (*P* < 0.001), higher levels of CA199 (*P* = 0.015), less signet ring cell carcinoma (*P* < 0.001), better differentiation (*P* = 0.017), and a larger proportion of women (*P* = 0.001), and less likely to receive adjuvant chemotherapy (*P* < 0.001) than the non-EGC group, even though the difference of gender, tumor grade, and chemotherapy coverage was only significant in the SEER cohort. However, in terms of location, Lauren type, stage, positive CEA rate, and the proportion receiving open surgery, both age groups did not differ significantly (*P* > 0.05).

### Survival analyses for OS and CSS

**Fig. 2:**
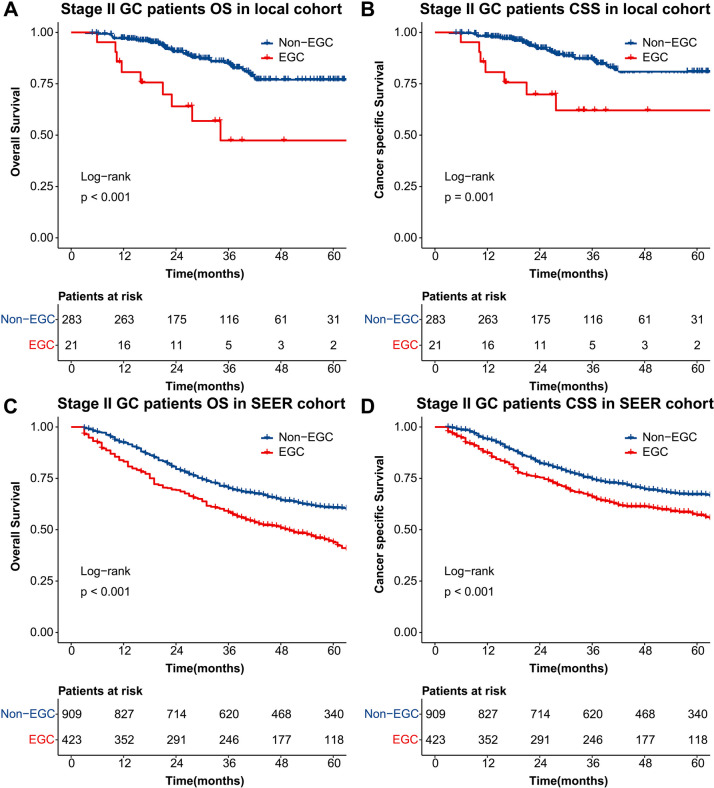
The Kaplan–Meier curves between age subgroups for overall survival and cancer-specific survival in stage II GC patients of local (**A**, **B**) and SEER cohorts (**C**, **D**)

In order to confirm the age partition established above, all patients in both cohorts were analyzed with Kaplan–Meier curves. In the local cohort, the OS rates of the EGC group were 80.7% at 1 year, 47.4% at 3 years, and 47.4% at 5 years, compared with 97.1% at 1 year, 85.1% at 3 years, and 77.0% at 5 years for the non-EGC group, respectively (*P* < 0.001 for the log-rank test) (Fig. 2A). In the SEER cohort, the OS rates of EGC group were 83.1% at 1 year, 58.5% at 3 years, and 43.8% at 5 years, compared with 92.3% at 1 year, 70.2% at 3 years, and 60.7% at 5 years for non-EGC group, respectively (*P* < 0.001 for the log-rank test) (Fig. 2C). Similarly, the CSS was evaluated in in both cohorts. In the local cohort, the CSS rates of the EGC group were 80.7% at 1 year, 62.1 at 3 years, and 62.1% at 5 years, compared with 98.2% at 1 year, 87.2 at 3 years, and 80.9% at 5 years for the non-EGC group, respectively (*P* = 0.001 for the log-rank test) (Fig. 2B). For the SEER participants, the CSS rates of the EGC group were 87.3% at 1 year, 65.9 at 3 years, and 57.1% at 5 years, compared with 94.0% at 1 year, 74.5 at 3 years, and 67.2% at 5 years for the non-EGC group, respectively (*P* < 0.001 for the log-rank test) (Fig. 2D).

### Univariate and multivariate Cox analyses

Furthermore, measures potentially associated with OS and CSS were analyzed using Cox proportional hazards regression methods. In the unadjusted analyses, the EGC group had a significantly poorer OS (hazard ratio (HR) = 3.77, 95% confidence interval (CI) 1.82–7.78, *P* < 0.001 for local cohort; HR = 1.78, 95% CI 1.52–2.09, *P* < 0.001 for SEER cohort) and CSS (HR = 3.50, 95% CI 1.54–7.93, *P* = 0.003 for local cohort; HR = 1.51, 95% CI 1.25–1.83, *P* < 0.001 for SEER cohort) whether in local or SEER cohort, respectively (Table 2). Further multivariate analyses demonstrated that advanced age was still a negative independent factor for OS (HR = 3.38, 95% CI 1.57–7.27, *P* = 0.002 for local cohort; HR = 1.50, 95% CI 1.26–1.79, *P* < 0.001 for SEER cohort) and CSS (HR = 3.74, 95% CI 1.64–8.55, *P* = 0.002 for local cohort; HR = 1.47, 95% CI 1.19–1.82, *P* < 0.001 for SEER cohort) in both cohorts. In addition, pathologically elevated CEA value, signet ring cell carcinoma, tumor evasion, lymph node status, and adjuvant chemotherapy were also independent predictors of OS and CSS, though the differences in some variates were not statistically significant in both cohorts (Table S1).

**Table 2:** Univariate and multivariate analyses of overall survival and cancer-specific survival in the local cohort

Characteristic	Overall survival				Cancer-specific survival			
	Univariate		Multivariate		Univariate		Multivariate	
	HR (95% CI)	*p* value^a^	HR (95% CI)	*p* value^a^	HR (95% CI)	*p* value^a^	HR (95% CI)	*p* value^a^
Age								
Non-EGC	1		1		1		1	
EGC	3.77 (1.82 − 7.78)	< 0.001	3.38 (1.57–7.27)	0.002	3.50 (1.54–7.93)	0.003	3.74 (1.64–8.55)	0.002
Gender								
Male	1				1			
Female	0.90 (0.49–1.66)	0.739			0.77 (0.39–1.55)	0.471		
BMI (kg/m^2^)								
18.5–24	1		1		1			
< 18.5	2.25 (1.13–4.49)	0.021	1.67 (0.81–3.45)	0.165	1.87 (0.85–4.12)	0.120		
≥ 24	0.67 (0.29–1.51)	0.331	0.70 (0.31–1.59)	0.396	0.55 (0.21–1.42)	0.215		
CEA								
Negative	1		1		1		1	
Positive	3.30 (1.84–5.91)	< 0.001	3.16 (1.74–5.74)	< 0.001	3.75 (1.99–7.07)	< 0.001	3.73 (1.96–7.11)	< 0.001
CA199								
Negative	1				1			
Positive	1.58 (0.74 − 3.36)	0.240			1.43 (0.6 − 3.4)	0.422		
Location								
Lower	1				1			
Upper	0.77 (0.38–1.58)	0.479			0.78 (0.36 − 1.71)	0.541		
Middle	1.36 (0.69 − 2.67)	0.370			1.21 (0.57 − 2.6)	0.616		
Overlapped	1.23 (0.29–5.30)	0.782			1.46 (0.33 − 6.38)	0.617		
Lauren type								
Intestinal	1				1			
Diffuse	1.27 (0.66–2.47)	0.477			1.72 (0.8–3.69)	0.168		
Mixed	1.19 (0.57–2.51)	0.640			1.50 (0.64–3.54)	0.354		
Grade								
Well	1				1			
Moderately	3.70 (0.49–27.9)	0.205			2.54 (0.33–19.67)	0.373		
Poorly and undifferentiated	3.97 (0.54–29.1)	0.175			3.43 (0.47–25.27)	0.226		
T stage								
T1-2	1				1		1	
T3-4	2.28 (0.82–6.35)	0.114	1.41 (0.40–4.98)	0.599	2.49 (0.77–8.06)	0.129	1.48 (0.32–6.73)	0.616
N stage								
N0	1				1		1	
N1	0.94 (0.52–1.69)	0.835	0.96 (0.51–1.8)	0.887	0.87 (0.45–1.67)	0.675	0.87 (0.43–1.75)	0.691
N2	0.24 (0.03–1.73)	0.156	0.33 (0.03–3.54)	0.361	0.28 (0.04–2.07)	0.213	0.42 (0.03–5.14)	0.493
N3	0 (0-Inf)	0.997	0 (0-Inf)	0.997	0 (0-Inf)	0.997	0 (0-Inf)	0.997
Surgery								
Open	1				1			
Laparoscope	1.05 (0.58–1.87)	0.879			0.97 (0.51–1.85)	0.937		
Chemotherapy								
No	1				1			
Yes	1.18 (0.59–2.36)	0.644			1.70 (0.71–4.06)	0.231		

*HR* hazard ratio, *CI* confidence interval

^a^Likelihood ratio tests

### Chemotherapy-based stratified analyses

**Fig. 3:**
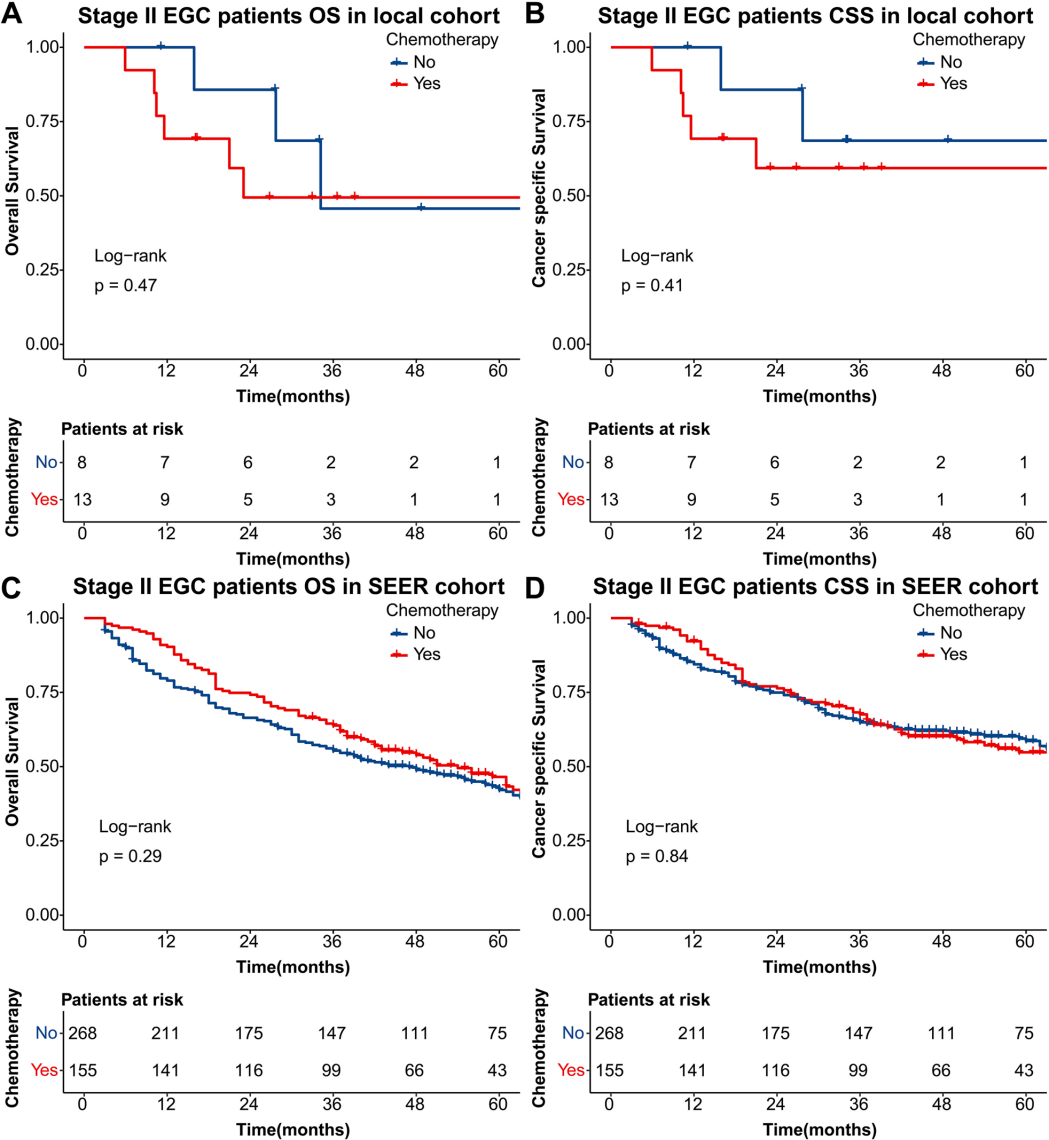
The Kaplan–Meier curves stratified by adjuvant chemotherapy for overall survival and cancer-specific survival in stage II EGC patients of local (**A**, **B**) and SEER cohorts (**C**, **D**)

Previous reports have still shown controversy about the efficacy and safety of adjuvant chemotherapy in stage II GC patients, even though recommended in clinical guidelines [10, 11]. Similarly, the abovementioned analyses showed divergences in the prognostic significance of chemotherapy. We considered that adjuvant chemotherapy should be more carefully evaluated for EGC patients. Therefore, we finally perform chemotherapy-stratified analyses in EGC patients in local and SEER cohorts. As shown in Fig. 3, in the local cohort, the differences in OS (Fig. 3A, *P* = 0.47) and CSS (Fig. 3B, *P* = 0.41) were not significant. Likewise, the differences in OS (Fig. 3C, *P* = 0.29) and CSS (Fig. 3D, *P* = 0.84) were also insignificant in the SEER cohort.

### External validation and exploration

**Fig. 4:**
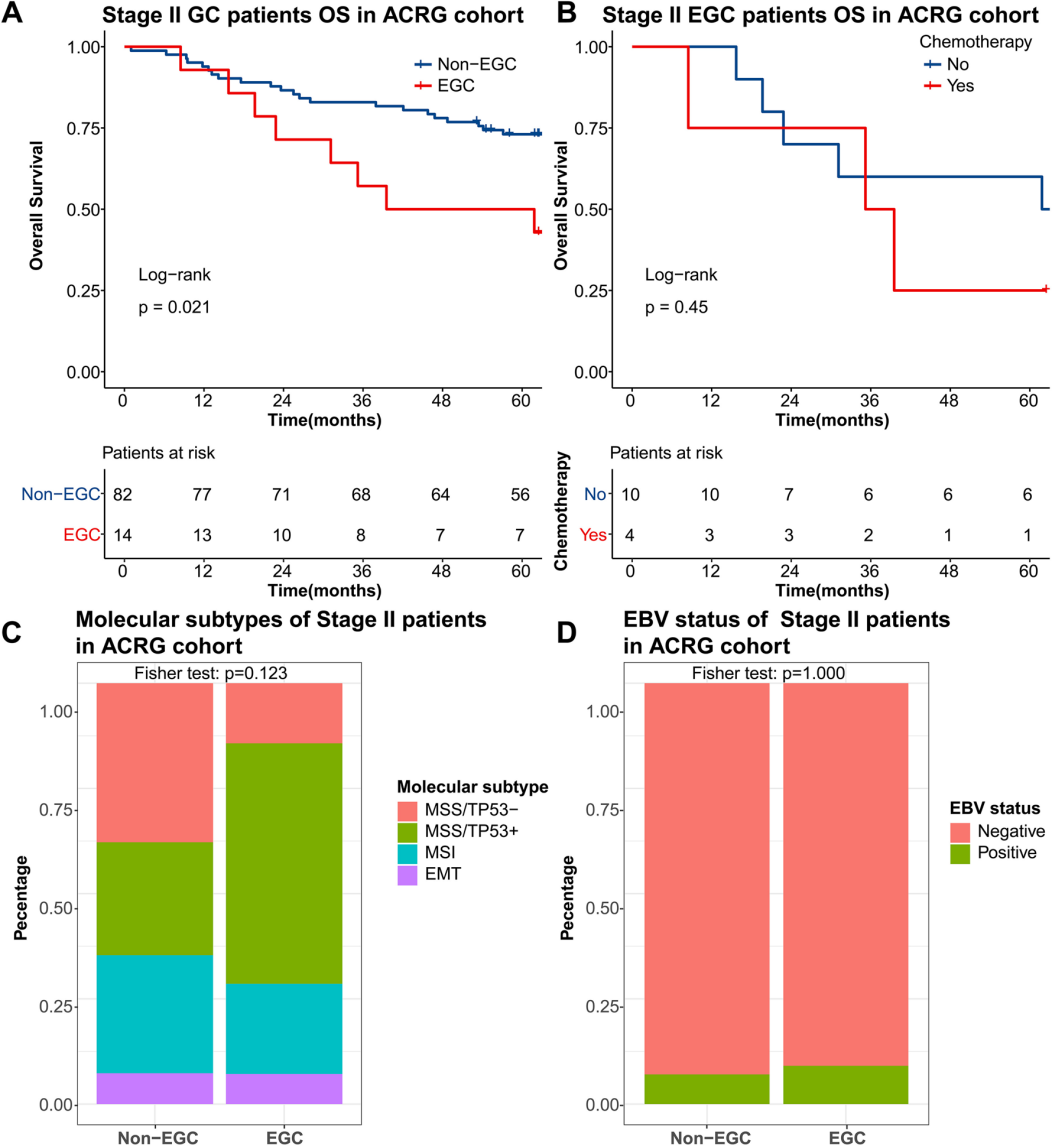
External validation and exploration in ACRG cohort. The age-stratified overall survival curve in stage II GC patients (**A**), and chemotherapy-stratified overall survival curve (**B**), molecular subtypes (**C**), and EBV status (**D**) of new-defined stage II EGC patients in ACRG cohorts

Finally, we validated the consistency of the preceding results in an external validation cohort, the ACRG cohort. Baseline characteristics of 96 stage II GC patients in the ACRG cohort are presented in Table S2, demonstrating no difference between EGC and non-EGC groups for all variables (*P* > 0.05). As expected, in the ACRG cohort, univariate (HR = 2.51, 95% CI 1.12–5.64, *P* = 0.026) and multivariate (HR = 2.76, 95% CI 1.21–6.32, *P* = 0.016) analyses demonstrated EGC was an independent risk factor. Moreover, the differences in OS (Fig. 4A, *P* = 0.021) between both groups were significant, while in the single EGC group, the differences in OS (Fig. 4B, *P* = 0.45) with and without chemotherapy were insignificant.

The inherent clinical heterogeneity is most likely due to differences in the molecular characteristics of cancer cells. We further investigated the molecular mechanism of poor prognosis in EGC patients and found that the molecular subtypes (Fig. 4C, *P* = 0.123 for Fisher’s test) and EBV status (Fig. 4D, *P* = 1.000 for Fisher’s test) between both groups did not reach statistically significant differences. However, further gene set variation analysis of transcriptome expression profiles revealed P53 pathway was negatively related (Fig. S3A, *R* =  − 0.26, *P* = 0.010) and the E2F targets pathway was positively correlated (Fig. S3B, *R* = 0.26, *P* = 0.010) with patient age. Similarly, the level of enrichment of DNA replication, mismatch repair, cell cycle, nucleotide excision repair, and homologous recombination pathway increased as diagnostic age increased (Fig. S3C).

## Discussion

Due to the peak incidence of gastric cancer predominantly occurring in the sixth decade of life, elderly patients occupy a large proportion. The burden of elderly gastric cancer will increase with aging worldwide. However, there is no consensus on the definition of EGC and the efficacy of adjuvant chemotherapy in patients with stage II EGC. According to some researchers, adjuvant chemotherapy could improve survival for the elderly [12, 13], but others did not find this to be the case [13–16]. Furthermore, chemotherapy can worsen the performance status of aged patients due to their poor tolerance of chemotherapeutic agents. Hence, we investigated the clinical efficacy of adjuvant chemotherapy in the defined EGC patient cohorts.

It was noteworthy to note that no standard definition of EGC exists. Previous researchers used artificial cutoff points to divide cases into groups (like 60 [10, 17], 70 [18, 19], 75 [20, 21], and 80 [22, 23] years old), then evaluated survival differences between the groups. Here, we performed the KAPS method to determine the most appropriate age partition value to define the EGC group. Based on survival data, heterogeneous subgroups could be created using this algorithm in an unsupervised way. The survival curves stratified by 75 years old both display good distinction for predicting survival in two derivation cohorts.

Subsequently, partitioned by the age of 75 years, the baseline characteristics of EGC and non-EGC patients showed that the EGC group exhibited lower BMI (*P* < 0.001), higher levels of CA199 level (*P* = 0.015), less signet ring cell carcinoma (*P* < 0.001), better differentiation (*P* = 0.017), and larger proportion of women (*P* = 0.001), and less likely to receive adjuvant chemotherapy (*P* < 0.001) than the younger counterparts.

The Kaplan–Meier survival analyses demonstrated that OS and CSS are particularly poor in EGC patients, whether in training cohorts or validation cohorts. Similarly, univariate and multivariate Cox proportional analyses in local, SEER, and ACRG cohorts recurrently demonstrated that advanced age was an independent predictor of OS and CSS in stage II GC patients who underwent curative resection. Finally, the effect of adjuvant chemotherapy was assessed in patients with stage II EGC. Chemotherapy-based stratified analyses indicated that the differences in OS and CSS in local and SEER cohorts between no chemotherapy and adjuvant chemotherapy groups did not reach a statistically significant difference, likewise in the external ACRG cohort.

Several studies have investigated the role of adjuvant chemotherapy on patients with stage II GC, and the results were inconsistent. In America, the Intergroup 0116 has demonstrated postoperative adjuvant chemotherapy provided survival benefits for stage II GC patients [24], and similar survival advantages were also found in the MAGIC and FLOT4 trials [25, 26]. In Eastern Asia, the ACTS-GC and CLASSIC trial also showed the OS and disease-free survival advantages of chemotherapy with S-1 or CAPOX. However, further subgroup analyses of these studies both showed no statistical prognostic significance of adjuvant chemotherapy for patients older than 60 or 70 years [5, 27].

Given the abovementioned conflicting results, a medical dilemma remains about whether to administer postoperative chemotherapy to EGC patients. This may be due to several reasons. First, due to shorter life expectancy and relatively limited lifespan, the elderly might not prefer adjuvant chemotherapy [28]. Second, these elderly patients would be more susceptible to treatment-related complications and comorbidity burden [29]. In addition, we interestingly noticed activation of DNA repair pathways and the down-modulation of p53 in the EGC patients in the GSVA of ACRG cohort, indicating that EGC patients received little benefit from adjuvant chemotherapy might attribute to the enhanced DNA repair pathways and down-modulation of p53 in EGC. Enhanced DNA repair pathways and down-modulating p53 in EGC patients can lead to resistance to DNA-damaging chemotherapeutic drugs [30, 31]. Tumor cells in EGC individuals become better at repairing drug-induced DNA damage, reducing the drugs’ effectiveness. Additionally, reduced p53 expression/activity weakens the cell response to DNA damage, making it harder for tumor cells to be affected by drugs like oxaliplatin [32]. This can result in poorer treatment outcomes or drug resistance. Further research is needed to understand these mechanisms better and develop personalized treatment strategies for EGC patients.

Until now, this is the first study to assess the real-world effects of adjuvant chemotherapy on the long-term survival of adjuvant chemotherapy among international multi-center EGC cohorts. However, in this study, several limitations were identified. Firstly, there is an inherent selection bias in this retrospective cohort study. Besides, medical records and public databases did not include detailed data such as comorbidities, complications, and chemotherapy details. Furthermore, the underlying mechanisms were responsible for these differences in molecular type between EGC and non-ECG. There is a need for further research on this critical issue.

## Conclusion

In this retrospective, international multi-center study, 75 years old was identified as the optimal age cutoff for EGC definition and reconfirmed its prognostic value in derivation and validation cohorts. We also investigated the adjuvant chemotherapy effectiveness in EGC patients and found that it did not provide survival benefits to stage II EGC patients.

### Supplementary Information

**Additional file 1:** **Fig. S1.** The flowchart of patient selection.

**Additional file 2:** **Fig. S2.** The KAPS scatter plot for age partitioning in local (A) and SEER cohorts (D) and Kaplan-Meier curves between age subgroups for overall survival and cancer-specific survival in local (B, C) and SEER cohorts (E, F).

**Additional file 3:** **Fig. S3.** Age-related gene set variation analyses in ACRG cohort. The scatter plot and Pearson correlation coefficients of age and P53 pathway or E2F targets in Hallmark collection, and enrichment score heatmap of age-related gene sets in KEGG collection.

**Additional file 4:** **Table S1.** Univariate and multivariate analyses of overall survival and cancer-specific survival in the SEER cohort.

**Additional file 5:** **Table S2.** The clinicopathological characteristics, univariate and multivariate analyses in the ACRG cohort.

## Data Availability

The datasets used and analyzed during the current study are available from the corresponding author on reasonable request.

## Abbreviations

ACRG: Asian Cancer Research Group
AJCC: American Joint Committee on Cancer
BMI: Body mass index
CA: Carbohydrate antigen
CEA: Carcinoembryonic antigen
CSS: Cancer-specific survival
EGC: Elderly gastric cancer
FOLFOX: Intravenous oxaliplatin, leucovorin, and 5-fluorouracil
GC: Gastric cancer
GEO: Gene Expression Omnibus
GSVA: Gene set variation analyses
KAPS: K-adaptive partitioning for survival data
NCCN: National Comprehensive Cancer Network
OS: Overall survival
SAH-SYSU: Sixth Affiliated Hospital of Sun Yat-sen University
SEER: Surveillance, Epidemiology, and End Results
SOX: Oral S-1 with intravenous oxaliplatin
XELOX: Oral capecitabine with intravenous oxaliplatin
