# Supplementary figures and images for: Elderly patients with stage II gastric cancer do not benefit from adjuvant chemotherapy
Source: World J Surg Oncol. 2023 Oct 11;21:319. doi: 10.1186/s12957-023-03185-5 (PMC10566074; doi:10.1186/s12957-023-03185-5)

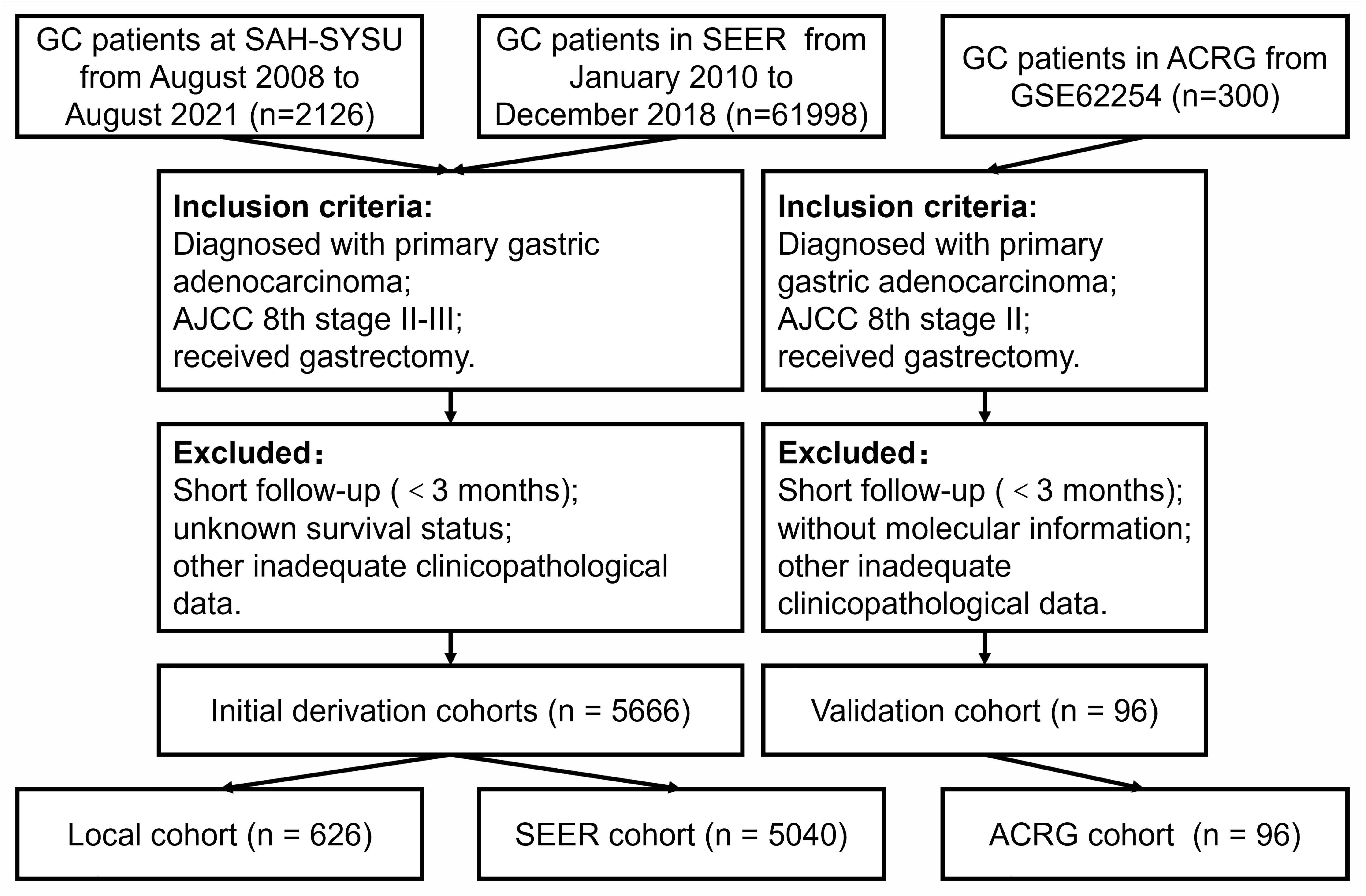

Supplement: Supplementary file 1 — Additional file 1: Fig. S1. The flowchart of patient selection. [file 12957_2023_3185_MOESM1_ESM.tif]

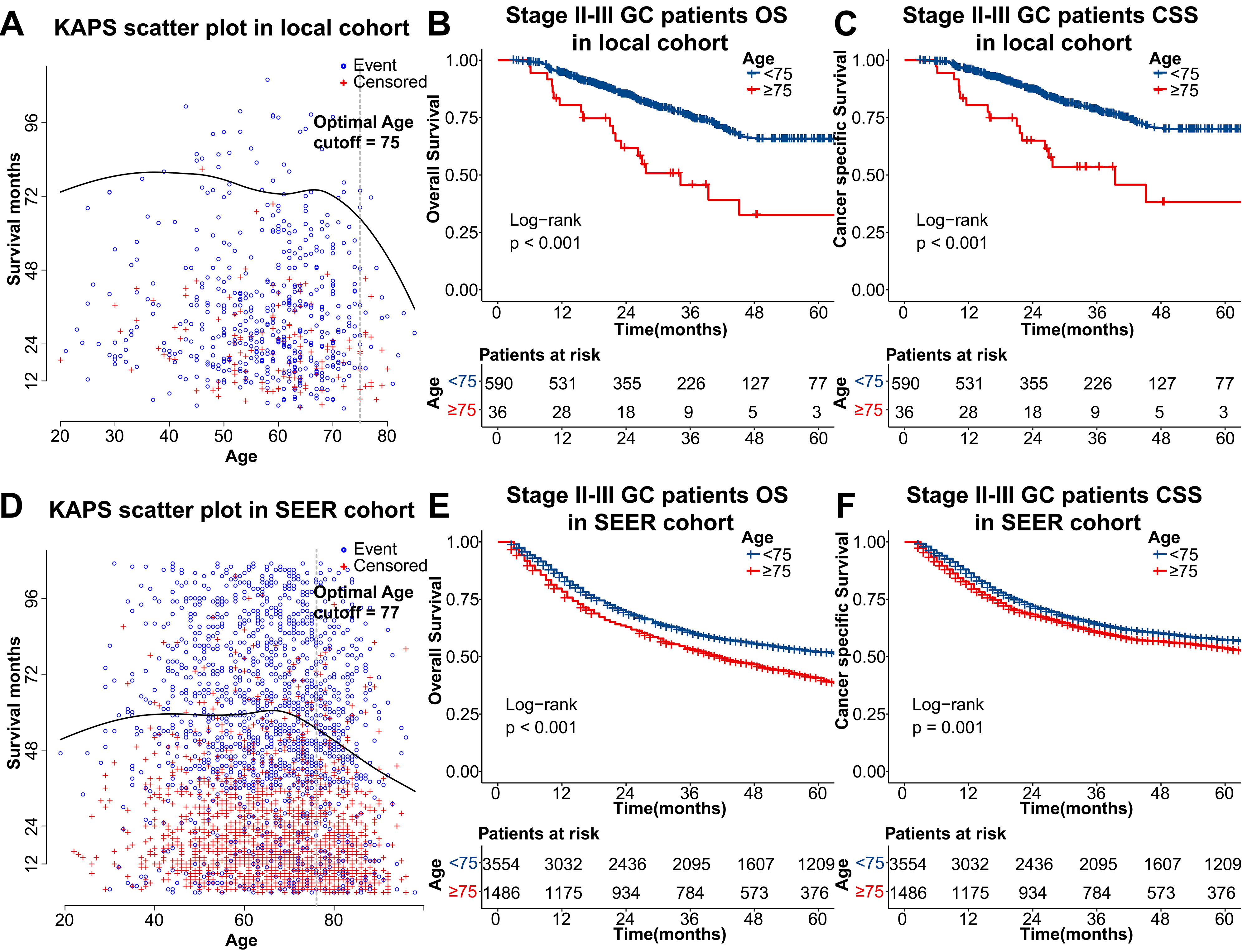

Supplement: Supplementary file 2 — Additional file 2: Fig. S2. The KAPS scatter plot for age partitioning in local (A) and SEER cohorts (D) and Kaplan-Meier curves between age subgroups for overall survival and cancer-specific survival in local (B, C) and SEER cohorts (E, F). [file 12957_2023_3185_MOESM2_ESM.tif]

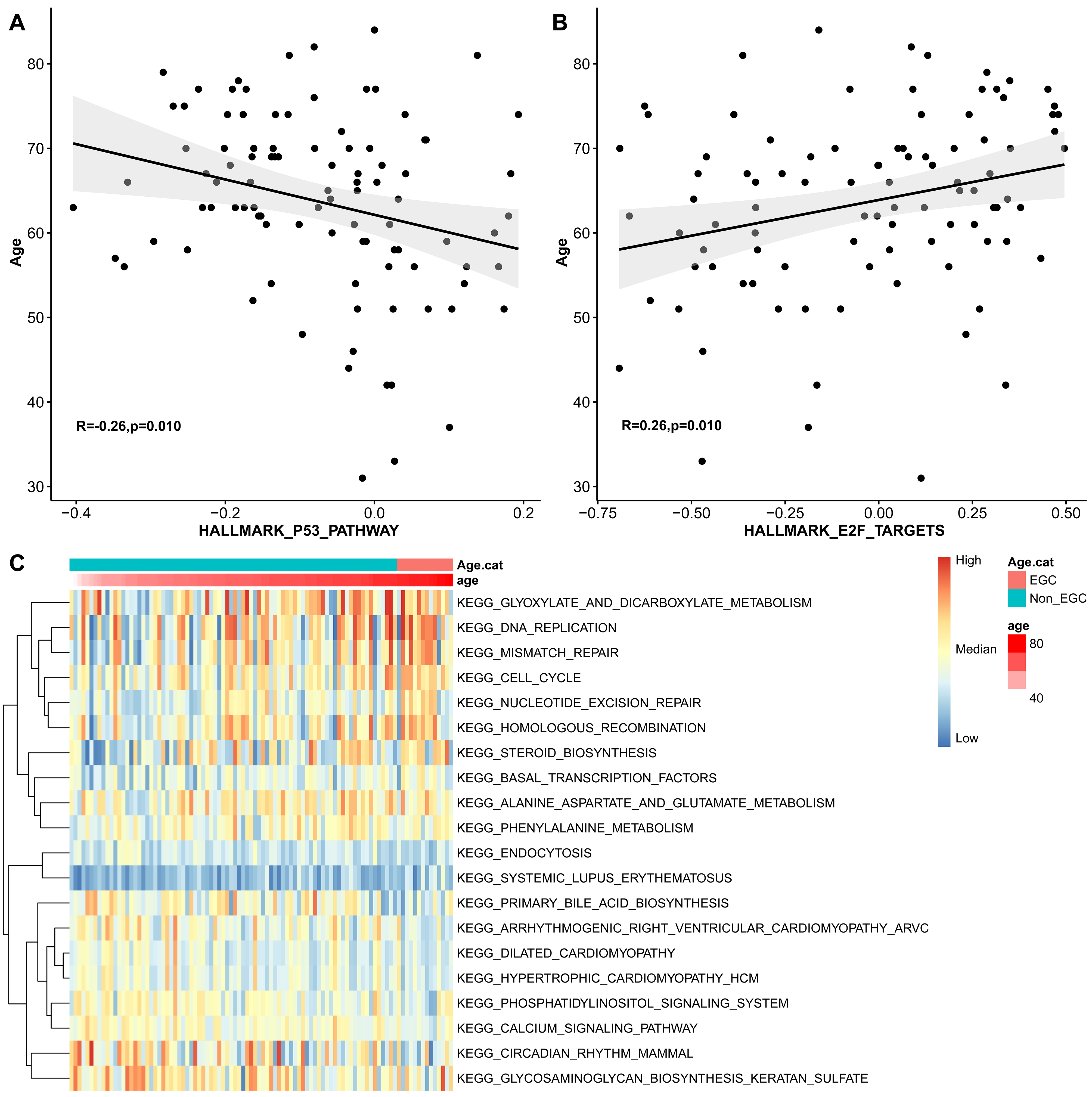

Supplement: Supplementary file 3 — Additional file 3: Fig. S3. Age-related gene set variation analyses in ACRG cohort. The scatter plot and Pearson correlation coefficients of age and P53 pathway or E2F targets in Hallmark collection, and enrichment score heatmap of age-related gene sets in KEGG collection. [file 12957_2023_3185_MOESM3_ESM.tif]
