# Supplementary material for: Elderly patients with stage II gastric cancer do not benefit from adjuvant chemotherapy
Source: World J Surg Oncol. 2023 Oct 11;21:319. doi: 10.1186/s12957-023-03185-5 (PMC10566074; doi:10.1186/s12957-023-03185-5)
Supplement: Supplementary file 4 — Additional file 4: Table S1. Univariate and multivariate analyses of overall survival and cancer-specific survival in the SEER cohort. [file 12957_2023_3185_MOESM4_ESM.docx]

**Table S1** Univariate and multivariate analyses of overall survival and cancer-specific survival in the SEER cohort.

| **Characteristic** | **Overall survival** | | | | |  | **Cancer-specific survival** | | | | |
| --- | --- | --- | --- | --- | --- | --- | --- | --- | --- | --- | --- |
|  | **Univariate** | |  | **Multivariate** | |  | **Univariate** | |  | **Multivariate** | |
|  | HR  (95% CI) | p-value ^a^ |  | HR  (95% CI) | p-value ^a^ |  | HR  (95% CI) | p-value ^a^ |  | HR  (95% CI) | p-value ^a^ |
| **Age** |  |  |  |  |  |  |  |  |  |  |  |
| Non-EGC | 1 |  |  | 1 |  |  | 1 |  |  | 1 |  |
| EGC | 1.78(1.52-2.09) | <0.001 |  | 1.50(1.26-1.79) | <0.001 |  | 1.51(1.25-1.83) | <0.001 |  | 1.47(1.19-1.82) | <0.001 |
| **Gender** |  |  |  |  |  |  |  |  |  |  |  |
| Male | 1 |  |  |  |  |  | 1 |  |  |  |  |
| Female | 1.11(0.95-1.31) | 0.177 |  |  |  |  | 1.16(0.96-1.39) | 0.122 |  |  |  |
| **Location** |  |  |  |  |  |  |  |  |  |  |  |
| Lower | 1 |  |  |  |  |  | 1 |  |  |  |  |
| Upper | 1.19(0.83-1.72) | 0.345 |  |  |  |  | 1.34(0.9-2) | 0.153 |  |  |  |
| Middle | 0.9(0.76-1.06) | 0.198 |  |  |  |  | 0.87(0.71-1.06) | 0.156 |  |  |  |
| Overlapped | 0.91(0.68-1.21) | 0.506 |  |  |  |  | 0.93(0.66-1.3) | 0.657 |  |  |  |
| **Histology** |  |  |  |  |  |  |  |  |  |  |  |
| Adenocarcinoma | 1 |  |  |  |  |  | 1 |  |  | 1 |  |
| Signet ring cell carcinoma | 1.09(0.91-1.32) | 0.347 |  |  |  |  | 1.26(1.02-1.56) | 0.029 |  | 1.50(1.2-1.86) | <0.001 |
| **Grade** |  |  |  |  |  |  |  |  |  |  |  |
| Well | 1 |  |  |  |  |  | 1 |  |  |  |  |
| Moderately | 1.28(0.77-2.13) | 0.338 |  |  |  |  | 1.12(0.62-2.02) | 0.713 |  |  |  |
| Poorly and undifferentiated | 1.35(0.82-2.23) | 0.234 |  |  |  |  | 1.4(0.79-2.49) | 0.253 |  |  |  |
| **T stage** |  |  |  |  |  |  |  |  |  |  |  |
| T1-2 | 1 |  |  | 1 |  |  | 1 |  |  | 1 |  |
| T3-4 | 1.29(1.06-1.56) | 0.010 |  | 1.57(1.18-2.08) | 0.002 |  | 1.17(0.94-1.46) | 0.149 |  | 1.63(1.19-2.24) | 0.003 |
| **N stage** |  |  |  |  |  |  |  |  |  |  |  |
| N0 | 1 |  |  | 1 |  |  | 1 |  |  | 1 |  |
| N1 | 1.16(0.98-1.37) | 0.082 |  | 1.50(1.25-1.80) | <0.001 |  | 1.39(1.14-1.69) | 0.001 |  | 1.74(1.4-2.15) | <0.001 |
| N2 | 0.87(0.66-1.16) | 0.343 |  | 1.46(0.98-2.17) | 0.063 |  | 1.00(0.72-1.38) | 0.998 |  | 1.71(1.09-2.7) | 0.021 |
| N3 | 0.97(0.52-1.82) | 0.922 |  | 1.68(0.84-3.35) | 0.143 |  | 1.29(0.66-2.52) | 0.454 |  | 1.96(0.93-4.11) | 0.077 |
| **Chemotherapy** |  |  |  |  |  |  |  |  |  |  |  |
| No | 1 |  |  | 1 |  |  | 1 |  |  | 1 |  |
| Yes | 0.58(0.50-0.68) | <0.001 |  | 0.64(0.53-0.76) | <0.001 |  | 0.74(0.62-0.90) | 0.002 |  | 0.75(0.60-0.92) | 0.007 |

HR: hazard ratio; CI: confidence interval.

^a^ Likelihood ratio tests.
