# Supplementary material for: Elderly patients with stage II gastric cancer do not benefit from adjuvant chemotherapy
Source: World J Surg Oncol. 2023 Oct 11;21:319. doi: 10.1186/s12957-023-03185-5 (PMC10566074; doi:10.1186/s12957-023-03185-5)
Supplement: Supplementary file 5 — Additional file 5: Table S2. The clinicopathological characteristics, univariate and multivariate analyses in the ACRG cohort. [file 12957_2023_3185_MOESM5_ESM.docx]

**Table S2** The clinicopathological characteristics, univariate and multivariate analyses in the ACRG cohort.

| **Characteristic** | **Baseline** | | |  | **Overall survival** | | | | |
| --- | --- | --- | --- | --- | --- | --- | --- | --- | --- |
|  |  |  |  |  | **Univariate** | |  | **Multivariate** | |
|  | Non-EGC  (N=82) | EGC  (N=14) | p-  value ^a^ |  | HR  (95% CI) | p-value ^b^ |  | HR  (95% CI) | p-value ^b^ |
| **Age** |  |  |  |  |  |  |  |  |  |
| Non-EGC |  |  |  |  | 1 |  |  | 1 |  |
| EGC |  |  |  |  | 2.51(1.12-5.64) | 0.026 |  | 2.76(1.21-6.32) | 0.016 |
| **Gender** |  |  | 1.000 |  |  |  |  |  |  |
| Male | 58 (70.7%) | 10 (71.4%) |  |  | 1 |  |  |  |  |
| Female | 24 (29.3%) | 4 (28.6%) |  |  | 0.55(0.23-1.35) | 0.193 |  |  |  |
| **Location** |  |  | 0.975 |  |  |  |  |  |  |
| Lower | 45 (54.9%) | 8 (57.1%) |  |  | 1 |  |  |  |  |
| Upper | 5 (6.1%) | 1 (7.1%) |  |  | 0.89(0.21-3.83) | 0.876 |  |  |  |
| Middle | 31 (37.8%) | 5 (35.7%) |  |  | 0.65(0.30-1.44) | 0.293 |  |  |  |
| Overlapped | 1 (1.2%) | 0 (0%) |  |  | 4.65(0.61-35.74) | 0.139 |  |  |  |
| **Lauren type** |  |  | 0.517 |  |  |  |  |  |  |
| Intestinal | 51 (62.2%) | 7 (50.0%) |  |  | 1 |  |  | 1 |  |
| Diffuse | 29 (35.4%) | 7 (50.0%) |  |  | 1.00(0.47-2.12) | 0.997 |  | 0.91(0.43-1.94) | 0.813 |
| Mix | 2 (2.4%) | 0 (0%) |  |  | 4.76(1.08-20.92) | 0.039 |  | 5.69 (1.27-25.43) | 0.023 |
| **T stage** |  |  | 0.904 |  |  |  |  |  |  |
| T1-2 | 78 (95.1%) | 14 (100%) |  |  | 1 |  |  |  |  |
| T3-4 | 4 (4.9%) | 0 (0%) |  |  | 0.83(0.11-6.08) | 0.853 |  |  |  |
| **N stage** |  |  | 0.766 |  |  |  |  |  |  |
| N0 | 5 (6.1%) | 0 (0%) |  |  | 1 |  |  |  |  |
| N1 | 77 (93.9%) | 14 (100%) |  |  | 1.60(0.22-11.77) | 0.642 |  |  |  |
| **Chemotherapy** |  |  | 0.089 |  |  |  |  |  |  |
| No | 35 (42.7%) | 10 (71.4%) |  |  | 1 |  |  |  |  |
| Yes | 47 (57.3%) | 4 (28.6%) |  |  | 0.57(0.28-1.16) | 0.120 |  |  |  |

HR: hazard ratio; CI: confidence interval.

^a^ Chi-square test or Fisher exact test.

^b^ Likelihood ratio tests.
